# Pharmacokinetics of Pantoprazole and Pantoprazole Sulfone in Goats After Intravenous Administration: A Preliminary Report

**DOI:** 10.3389/fvets.2021.744813

**Published:** 2021-09-22

**Authors:** Joe S. Smith, Jonathan P. Mochel, Windy M. Soto-Gonzalez, Rebecca R. Rahn, Bryanna N. Fayne, Olivia G. Escher, Anastasia M. Geletka, Lainey E. Harvill, Joan B. Bergman, Sherry Cox

**Affiliations:** ^1^Systems Modeling and Reverse Translational Pharmacology, Department of Biomedical Sciences, College of Veterinary Medicine, Iowa State University, Ames, IA, United States; ^2^Department of Large Animal Clinical Sciences, College of Veterinary Medicine, University of Tennessee, Knoxville, Knoxville, TN, United States; ^3^Department of Biomedical and Diagnostic Sciences, College of Veterinary Medicine, University of Tennessee, Knoxville, Knoxville, TN, United States

**Keywords:** goat (capra aegagrus hircus), pantoprazole, pantoprazole sulfone, pharmacokinetics, ulcer

## Abstract

**Background:** Ruminant species are at risk of developing abomasal ulceration, but there is a lack of pharmacokinetic data for anti-ulcer therapies, such as the proton pump inhibitor pantoprazole, in goats.

**Objective:** The primary study objective was to estimate the plasma pharmacokinetic parameters for pantoprazole in adult goats after intravenous administration. A secondary objective was to describe the pharmacokinetic parameters for the metabolite, pantoprazole sulfone, in goats.

**Methods:** Pantoprazole was administered intravenously to six adult goats at a dose of 1 mg/kg. Plasma samples were collected over 36h and analyzed via reverse phase high performance liquid chromatography for determination of pantoprazole and pantoprazole sulfone concentrations. Pharmacokinetic parameters were determined by non-compartmental analysis.

**Results:** Plasma clearance, elimination half-life, and volume of distribution of pantoprazole were estimated at 0.345 mL/kg/min, 0.7 h, and 0.9 L/kg, respectively following IV administration. The maximum concentration, elimination half-life and area under the curve of pantoprazole sulfone were estimated at 0.1 μg/mL, 0.8 h, and 0.2 hr^*^μg/mL, respectively. The global extraction ratio was estimated 0.00795 ± 0.00138. All animals had normal physical examinations after conclusion of the study.

**Conclusion:** The reported plasma clearance for pantoprazole is lower than reported for foals, calves, and alpacas. The elimination half-life appears to be < that reported for foals and calves. Future pharmacodynamic studies are necessary for determination of the efficacy of pantoprazole on acid suppression in goats.

## Introduction

Pantoprazole is a member of the proton pump inhibitor (PPI) class of gastro protectant drugs that functions by irreversibly binding to the hydrogen-potassium-ATPase pump in gastric parietal cells, resulting in reduced gastric acid secretion and increased gastric pH (1, 2). In veterinary species, the PPIs have been recognized as the most potent suppressors of gastric acid (3). Drugs of the PPI class such as omeprazole have been thoroughly investigated as a treatment for gastric ulceration in horses (4). Abomasal ulceration is the manifestation of gastric ulceration in ruminant species (5), which can be caused by disease, stress, or as an adverse effect of some medications, such as nonsteroidal anti-inflammatory drugs. Reports in the literature of pharmacologic options for the management of this disease in ruminant species is currently limited.

Abomasal ulcerations range in severity from non-perforating to perforating with diffuse peritonitis in ruminant species (5). Multiple therapies have been described for ulceration in other veterinary species, including coating agents, sucralfate, histamine type two receptor antagonists, as well as PPIs (3). One of the challenges with gastro protectant therapy in mature ruminants is the rumen's ability to dilute and degrade orally administered medications. As such, parenteral administration provides a route of bypassing the barriers posed by the rumen for oral therapies. Famotidine has been described for use in mature steers, but it requires multiple daily administrations and had diminishing effects over time (6). Omeprazole has been described in some mature psuedoruminant species, such as llamas (7, 8) but the injectable formulation is not available globally, and other routes of administration, such as oral and rectal appear to lead to poor absorption (8, 9). Pantoprazole is commonly used in human medicine, is readily available, and as such may be a useful therapy for goats at risk for ulceration.

The use of pantoprazole has been describe in multiple ruminant species including cattle, alpacas, sheep, goats, yaks, and camels (10–17). Most of these uses are focused on increasing the abomasal luminal pH to create an environment conducive to the healing of gastric ulcers. Among large animal species, the pharmacokinetics of pantoprazole has only been reported in alpacas, neonatal calves, and foals (10, 11, 18). The primary objective of this study was to determine the pharmacokinetics of pantoprazole in adult goats after single intravenous (IV) administration. A secondary objective of this study was to evaluate the pharmacokinetics of the metabolite pantoprazole sulfone, after IV administration of pantoprazole in adult goats.

## Materials and Methods

The study protocol was reviewed and approved by the Institutional Animal Care and Use Committee of the University of Tennessee (Protocol # 2825-0221). Six healthy adult goats were utilized for this study. Three goats were pygmies and three were pygmy-crosses. Ages were 3.2 ± 0.7 years and weights were 42.1 ± 6.1 kgs. Four of the goats were intact females, and two were castrated males. Goats were sourced from the teaching herd of the Veterinary Research and Education Center of the University of Tennessee. During the study they were fed a diet of *ad libitum* grass hay. None of the animals had been medicated within the 4 weeks prior to the study and all were current on vaccination for Clostridium perfringens types C and D, as well as tetanus. Before employment for the study all goats were deemed healthy based on physical examination by large animal veterinary specialist. All goats had an intravenous catheter aseptically placed into each jugular vein as previously reported, with one catheter designated for blood collection and another one utilized for drug administration (11, 19). Pantoprazole was reconstituted to a 4 mg/mL concentration per label instruction. A 1 mg/kg dosage of pantoprazole (Pantoprazole sodium for injection, AuroMedics Pharma LLC, East Windsor, NJ) was administered intravenously to each goat. Blood samples were collected at 0, 10, 20, 30, and 45 min after collection as well as 1, 1.5, 2, 3, 4, 8, 12, 18, 24, and 36 h after drug administration. Blood samples were placed into a lithium heparin tubes after collection, and then immediately spun down and transferred to cryogenic vials placed into storage at −80°C until analysis.

### Analytical Method

Analysis of pantoprazole in plasma samples was conducted using reverse phase high performance liquid chromatography method. The system consisted of a computer equipped with Empower software (Waters), a 2,695 separations module, and a 2,487 UV absorbance detector. The compounds were separated on a Symmetry C_18_ (4.6 x 150 mm, 5μm) column with a 5μm Symmetry C_18_ guard column. The mobile phase was a mixture of 0.1 M sodium phosphate dibasic (14.2857 g Na_2_HPO_4_) and acetonitrile (68:32). The flow rate was 1 mL/min and absorbance was measured at 290nm.

Pantoprazole and the metabolite were extracted from plasma samples using a liquid-liquid extraction method. Samples that were previously frozen were thawed, vortex-mixed, and 100μl of plasma was transferred to a 13 x 100mm screw top tube followed by 10μl of tinidazole (internal standard, 100 μg/mL) and 2 mL chloroform. The tubes were rocked for 15 min and then centrifuged for 20 min at 1,000 x g. The organic layer was transferred to a clean tube and evaporated to dryness with nitrogen gas. Samples were reconstituted in 250μL of mobile phase and 100μL was analyzed.

Standard curves for the plasma analysis were prepared by fortifying untreated, pooled plasma with pantoprazole and its metabolite, which produced a linear concentration range of 0.01–100 μg/mL. Methanol was used as a solvent. Calibration samples were prepared the same as the plasma samples. Average recovery for pantoprazole and its metabolite was 100 and 90%, respectively. The average recovery for the internal standard was 99%. The QC samples used for validation were 0.03, 0.3, 3, and 30 μg/mL and the intra and inter-assay variability ranged from 2 to 11 % for pantoprazole and 3– 9% for the metabolite. The lower limit of quantification for both was 0.1 μg/mL.

### Pharmacokinetic Analysis

Pharmacokinetic parameters for pantoprazole systemic disposition were determined from plasma time vs. concentration data as previously described by Olivarez et al. (11). Pharmacokinetic analysis was performed via commercial modeling software using a statistical moments approach (PKanalix, Monolix Suite 2020R1, Lixoft, France). Standard time vs. concentration data for pantoprazole were determined via reverse phase high performance liquid chromatography from the blood collected at 15 time points ranging from 0 to 36 h after administration.

Standard PK parameters were generated for individual goats, as follows:

1. Maximum concentration extrapolated to time zero, C0 (pantoprazole);
2. Area under pantoprazole concentration–time curve, AUClast and AUCinf;
3. Area under the moment curve, AUMCinf;
4. Pantoprazole mean residence time, MRT = AUMCinf/AUCinf;
5. Pantoprazole terminal half-life, T1/2 (λz)) = ln (2)/λz;
6. Pantoprazole systemic clearance, CL = Dose/AUCinf;
7. Volume of distribution of pantoprazole at steady-state, Vss = CL × MRT

For data analysis, a log trapezoidal rule was used to estimate the area under the pantoprazole time-curves. Summary statistics on the individual PK parameters were performed thereafter to derive the geometric mean, median and (min–max) range.

Global extraction ratio (Ebody) was first calculated as reported by Toutain and Bousquet-Melou (20), with:


$$\begin{array}{l} Ebody=Systemic\;clearance/Cardiac\;output \end{array}$$

First calculated for each individual goat, and then combined for a mean value. With the goat cardiac output calculated as previously reported (20, 21) as follows:


$$\begin{array}{l} Cardiac\;output=180\;\times BW{\left(kg\right)}^{-0.19} \end{array}$$

Pharmacokinetic parameters for the metabolite, pantoprazole sulfone were determined from plasma time vs. concentration data as previously described for norfentanyl (22). Parameters described are: maximum concentration; time to maximum concentration; area under the curve (at last measurement and extrapolated to infinity); area under the moments curve; elimination half-life; and mean residence time. Pharmacokinetic modeling was performed via commercial modeling software (PKanalix, Monolix Suite 2020R1, Lixoft, France). Standard time vs concentration data for pantoprazole sulfone were determined via reverse phase high performance liquid chromatography from the blood collected at 15 time points ranging from 0 to 36h after administration.

## Results

**Figure 1:**
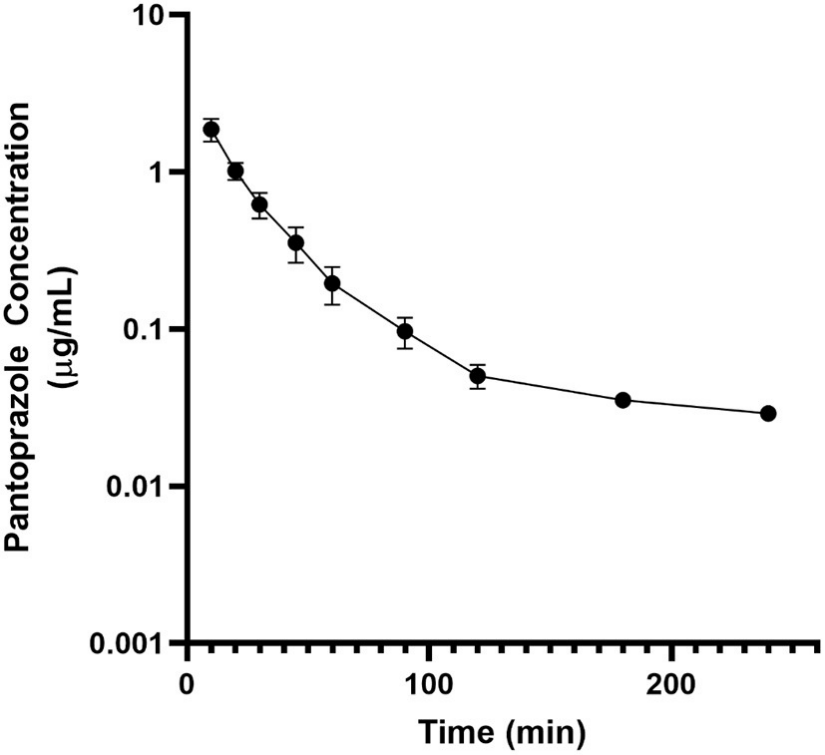
Mean plasma pantoprazole concentration (logarithmic scale) vs. time (hr) profiles for adult goats (*n* = 6) following intravenous (IV) single dose administration of 1.0 mg/kg of pantoprazole. Mean is represented by a black circle with error bars.

No adverse effects were observed in any of the goats used for this study. No animal displayed altered appetite or attitude, and no edema or signs of anaphylaxis were observed. No concentrations of pantoprazole were detected after 4h in any animal. Table 1 displays the geometric mean, median, minimum and maximum of the pharmacokinetic parameters of pantoprazole for the goats in our study. The elimination half-life, V_z_ and AUC were (arithmetic mean ± standard deviation) were: 0.7 ± 0.4 (hr); 1.1 ± 0.9 (L/kg); and 1.2 ± 0.4 (hr^*^μg/mL), respectively. Figure 1 presents the time vs. concentration curve for pantoprazole. Mean extraction ratio for pantoprazole was 0.795 ± 0.138 %.

**Table 1:** Pantoprazole pharmacokinetic parameters following a single intravenous (1 mg/kg) administration to adult goats.

**Compound**	**Parameter**	**Unit**	**Geomean**	**Median**	**Min**	**Max**
Pantoprazole	C0	μg/mL	3.1	4.1	1.0	5.9
	AUC_last_	hr*μg/mL	1.1	1.1	0.7	1.8
	AUC_inf_	hr*μg/mL	1.1	1.2	0.8	1.8
	AUMC_inf_	hr^2^*μg/mL	0.6	0.7	0.3	1.0
	MRTI_NF_	hr	0.5	0.5	0.4	0.9
	Cl	mL/kg/hr	20.5	20.5	13.3	31.4
	T_1/2_ (λz)	hr	0.7	0.6	0.5	1.5
	V_ss_	L/kg	0.5	0.5	0.3	1.2
	Vz	L/kg	0.9	0.8	0.4	2.7

*AUClast, Area under pantoprazole concentration–time curve from time zero to last measurement; AUCinf, Area under pantoprazole concentration–time curve from time zero to infinity; AUMCinf, the area under the first-moment curve from time zero to infinity; MRTinf, Mean residence time extrapolated to infinity; Cl, Plasma clearance; T1/2 (λz), Elimination half-life; C0, Maximum concentration extrapolated to time zero; Cmax, Maximum concentration; Vz, Volume of distribution (terminal phase); Vss, Volume of distribution at steady-state*.

**Figure 2:**
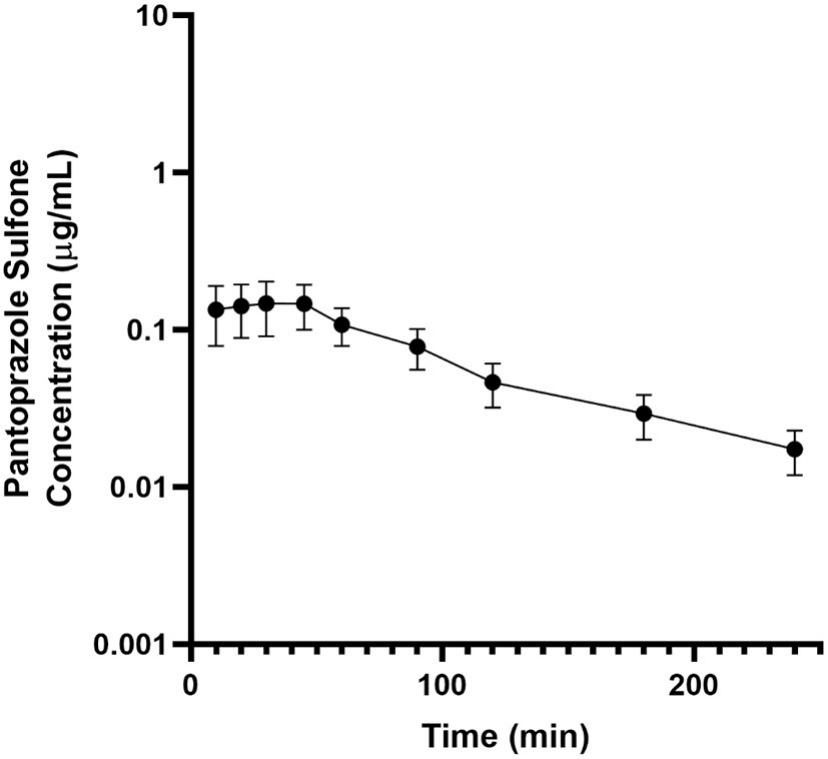
Mean plasma pantoprazole sulfone concentration (logarithmic scale) vs. time (hr) profiles for adult goats (*n* = 6) following intravenous (IV) single dose administration of 1.0 mg/kg of pantoprazole. Mean is represented by a black circle with error bars.

Table 2 displays the geometric mean, median, minimum and maximum of the pharmacokinetic parameters of the metabolite pantoprazole sulfone within the goats of our study. No concentrations of pantoprazole sulfone were detected after 4h in any animal. Figure 2 presents the time vs. concentration curve for pantoprazole sulfone.

**Table 2:** Pantoprazole sulfone pharmacokinetic parameters following a single intravenous (1 mg/kg) administration of pantoprazole sodium to adult goats.

**Compound**	**Parameter**	**Unit**	**Geomean**	**Median**	**Min**	**Max**
Pantoprazole	C_max_	μg/mL	0.1	0.2	0.04	0.4
Sulfone	T_max_	hr	0.5	0.5	0.3	0.8
	AUC_last_	hr*μg/mL	0.2	0.2	0.1	0.6
	AUC_inf_	hr*μg/mL	0.2	0.2	0.1	0.7
	AUMC_inf_	hr^2^*μg/mL	0.3	0.3	0.1	0.9
	T_1/2_ (λz)	hr	0.8	0.9	0.5	1.0
	MRTI_NF_	hr	1.3	1.4	1.1	1.6

*Cmax, Maximum concentration; Tmax, Time to maximum concentration; AUClast, Area under pantoprazole concentration–time curve from time zero to last measurement; AUCinf, Area under pantoprazole concentration–time curve from time zero to infinity; AUMCinf, the area under the first-moment curve from time zero to infinity; T1/2 (λz), Elimination half-life; MRTinf, Mean residence time extrapolated to infinity*.

## Discussion

When administered intravenously at 1 mg/kg as a single dose, pantoprazole is characterized by rapid elimination in goats compared to other veterinary species. The elimination half-life of pantoprazole in the goats in our study was (mean of 0.7 hr) was < observed in foals (1.4 hr) and calves (2.8 hr), however it was longer than the observed elimination half-life in alpacas (0.5 hr) (10, 11, 18). Clearance (mean) in our goats (20.5 mL/kg/hr, approximately 0.341 mL/kg/min) was lower than reported for foals (1.3 mL/kg/min), calves (4.5 mL/kg/min), or alpacas (12.2 mL/kg/min)(10, 11, 18). This is an interesting finding as the rapid elimination half-life could be consistent with an increased relative clearance value. However, half-life is a hybrid parameter, which is a function of clearance and distribution volume, so it is possible that interspecies differences in volume of distribution could contribute to the differences in parameters. In addition to species-specific differences, it is also possible that analytical sensitivity could contribute to differences in parameters. The observed extraction ratio was lower (0.00795) than reported for calves (0.053). It is possible that these differences could be due to species-specific variances of pantoprazole metabolism, or potentially as a factor of age, as the goats in this study were adults, and the previous studies of foals and calves utilized neonates. Goats, due to their selective browse grazing behavior are thought to have more robust enzyme systems for metabolizing xenobiotic substances than non-specific grazers such as cattle (23), so these differences compared to calves could also be species-specific.

There is currently a paucity of information regarding the disposition of the metabolites of pantoprazole in veterinary species. In calves, pantoprazole sulfone has been detected in tissues 5 days after administration. In that study, no levels of the parent drug pantoprazole were detected in tissue at any time (11). In dogs, two metabolites of pantoprazole have been detected after intravenous administration, pantoprazole sulfone and pantoprazole thioether (24). In humans, pantoprazole sulfone is used in pharmacokinetic studies, and population variation in pharmacokinetics has been observed, particularly in obese children (25, 26). In obese children, a variation of 3–5 times normal parameters had been observed, and it has been recommended to avoid empiric dosage escalation in these patients (25). Pantoprazole is thought to be metabolized in the liver by CYP2C19 enzymes, with the majority of the drug excreted in the urine.

In human patients, several adverse effects have been attributable to pantoprazole including anaphylaxis, thrombocytopenia and electrolyte disorders (27–31). While uncommon, these adverse effects also appear to be limited to geriatric human patients that undergo long-term therapy with pantoprazole (31). Pancreatitis and peripheral edema have also been reported in human patients treated with pantoprazole (32–34). A case of pantoprazole-induced hyperthermia was reported in a human patient, however, none of our study goats had elevated temperatures during the study (35). While an investigation of adverse effects was beyond the scope of our present study, no gross adverse effects, such as edema or anaphylaxis were observed in this population. This is consistent with a retrospective study investigating the safety of pantoprazole in hospitalized ruminants (12), as well as several cases reported in the literature of the use of pantoprazole in goats (13, 14, 36), although future safety investigations are necessary to completely capture the safety profile of this drug in caprine patients.

Limitations of this study include its small sample size. However, many veterinary pharmacokinetic studies employ six animals, and studies of four–six animals are typically adequate for describing pharmacokinetics (37). An additional limitation lies in the rapid nature of the elimination of pantoprazole in the goat, which combined with the study sampling schedule, could potentially not allow a thorough characterization of the elimination phase. Future studies may want to consider higher dosages or potentially extravascular administration, such as subcutaneous administration to prolong drug exposure, or increasing the frequency of early sampling. An additional limitation is the inability to provide a complete pharmacokinetic profile for pantoprazole sulfone, as at this time, the complete metabolism of pantoprazole in the goat is unknown. Due to the potential genetic nature of CYP2C19 enzyme activity, the small population of animals could be a limitation, although all of the study goats were unrelated, and they represented several different breed crosses. Future studies to investigate the metabolism of pantoprazole in the goat will be needed to determine the ratios of and specific metabolites.

This study lays the foundation for multiple lines of work. Future endeavors will need to investigate the pharmacodynamics and efficacy of pantoprazole in goats to determine if the observed increased elimination compared to other species results in a decreased efficacy of the therapeutic drug. Pantoprazole is thought to have activity beyond its duration in the system due to the irreversible nature of its binding at the proton pump. It is unknown if a specific concentration needs to be achieved for a specific time period for the desired effect. Of specific interest would be the correlation between area under the curve and clinical efficacy. Currently, the epigenetic potential of pantoprazole has not been investigated in any species, and it is unknown if pantoprazole may have any epigenetic effects or quasi-epigenetic effects such as other receptor down- or up-regulation (38). Additional studies could utilize nonlinear mixed effects modeling to evaluate variability in pantoprazole pharmacokinetics (39). Further studies will also have to determine the effect of pantoprazole on the gastrointestinal microbiome resulting from the potential changes in gastrointestinal pH. Finally, as the use of pantoprazole would be considered extra-label usage (14), future studies to determine withdrawal intervals will be necessary in goats.

In conclusion, pantoprazole administered by intravenous injection appears to be well tolerated and quickly eliminated in adult goats. Plasma clearance appears to be reduced and elimination half-life appear to be faster than previous reports in foals, calves, and alpacas. Pantoprazole sulfone was detectable in the plasma for up to 4h after administration, although at lower concentrations than the parent drug. Future studies in larger study populations are required to completely elucidate the pharmacokinetics and efficacy of pantoprazole in goats.

## Data Availability Statement

The original contributions presented in the study are included in the article/supplementary material, further inquiries can be directed to the corresponding author/s.

## Ethics Statement

The animal study was reviewed and approved by Institutional Animal Care and Use Committee, University of Tennessee.

## Author Contributions

JS, JM, and SC developed the experimental design. WS-G, RR, BF, OE, AG, and LH contributed to logistical support, assessment of animal health, and data collection. LH, SC, and JB developed the analytical method for determination of concentration. JS and JM performed the pharmacokinetic analysis. All authors contributed to manuscript construction.

## Conflict of Interest

The authors declare that the research was conducted in the absence of any commercial or financial relationships that could be construed as a potential conflict of interest.

## Publisher's Note

All claims expressed in this article are solely those of the authors and do not necessarily represent those of their affiliated organizations, or those of the publisher, the editors and the reviewers. Any product that may be evaluated in this article, or claim that may be made by its manufacturer, is not guaranteed or endorsed by the publisher.
